# Psychopathology and treatment of Prader-Willi syndrome in adulthood: About a clinical case

**DOI:** 10.1192/j.eurpsy.2021.1914

**Published:** 2021-08-13

**Authors:** G.M. Ruiz Martinez, A. Alvarado Dafonte, L. Soldado Rodriguez

**Affiliations:** Mental Health Unit, Complejo Hospitalario de Jaen, Jaen, Spain

**Keywords:** Treatment, genetics, prader-willi, PSYCHOPATOLOGY

## Abstract

**Introduction:**

Prader-Willi syndrome is a congenital disease caused by a genetic alteration of chromosome 15, described by doctors Prader, Labhart and Willi in 1956. It’s a rare disease (prevalence 2.8/100,000) and it occurs equally in both sexes and in all races. This disorder produces changes in hypothalamic function that can lead to muscle hypotonia, short stature, a compulsion to eat, and a lack of satiety. At the psychopathological level, the clinic is very varied, being mainly important the psychomotor retardation in different degrees and behavioral problems;especially in the behavioral phenotype. Affective and psychotic symptoms are also frequent.

**Objectives:**

Psychopathology and treatment analysis through a clinical case.

**Methods:**

40-year-old patient undergoing mental health follow-up since adolescence with a diagnosis of paranoid personality disorder. No medical history of interest. He was admitted to the hospitalization unit for serious behavioral alterations in a context of probable paranoid ideation towards the neighborhood. In the psychopathological examination, marked cognitive rigidity, high impulsivity and very low tolerance to frustration stand out, showing a hostile and defiant attitude. Poorly structured paranoid ideation. Presents obsessive-compulsive behaviors (scratching). Hyperphagia and obesity.

**Results:**

Psychometric assessment (Waiss-IV): total IQ 61 (mild mental retardation). Genetics:deletion 15 q11-q13 of chromosome 15), confirms Prader-Willi diagnosis. Remission of behavioral disorders, suspiciousness and heteroaggressive behaviors with treatment with monthly depot paliperidone (150 mg).

**Conclusions:**

Knowledge of the clinical and morphological characteristics of this syndrome would allow an early diagnosis and treat its possible complications as soon as possible. Antipsychotic treatment is effective in the management of behavioral and psychotic symptoms.

**Disclosure:**

No significant relationships.

